# A scoping review of randomized trials assessing the impact of n-of-1 trials on clinical outcomes

**DOI:** 10.1371/journal.pone.0269387

**Published:** 2022-06-02

**Authors:** Joyce P. Samuel, Susan H. Wootton, Travis Holder, Donald Molony

**Affiliations:** 1 Center for Clinical Research and Evidence-Based Medicine, McGovern Medical School, The University of Texas Health Science Center at Houston, Houston, TX, United States of America; 2 Houston Academy of Medicine, The Texas Medical Center Library, Houston, TX, United States of America; Thomas Jefferson University, UNITED STATES

## Abstract

**Background:**

The single patient (n-of-1) trial can be used to resolve therapeutic uncertainty for the individual patient. Treatment alternatives are systematically tested against each other, generating patient-specific data used to inform an individualized treatment plan. We hypothesize that clinical decisions informed by n-of-1 trials improve patient outcomes compared to usual care. Our objective was to provide an overview of the clinical trial evidence on the effect of n-of-1 trials on clinical outcomes.

**Methods:**

A systematic search of medical databases, trial registries, and gray literature was performed to identify trials assessing clinical outcomes in a group of patients undergoing an n-of-1 trial compared to those receiving usual care for any clinical condition. We abstracted elements related to study design and results and assessed risk of bias for both the overall randomized trials and the n-of-1 trials. The review was registered on PROSPERO. (CRD: 42020166490).

**Findings:**

Twelve randomized trials of the n-of-1 approach were identified in conditions spanning chronic pain, osteoarthritis, chronic irreversible airflow limitation, attention-deficit hyperactivity disorder, hyperlipidemia, atrial fibrillation, statin intolerance, and hypertension. One trial showed a statistically significant benefit in the primary outcome. Only one reached the pre-specified sample size target. Secondary outcomes showed modest benefits, including decreasing medication use, fewer atrial fibrillation episodes, and improved patient satisfaction.

**Interpretation:**

Very few trials have been undertaken to assess the effectiveness of n-of-1 trials in improving clinical outcomes, and most trials were underpowered for the primary outcome. Barriers to enrollment and retention in these trials should be explored, as well-powered randomized trials are needed to clarify the clinical impact of n-of-1 trials and assess their utility in clinical practice.

## Introduction

N-of-1 trials are single patient randomized trials that can provide direct and objective assessments of treatment effects in the individual patient and can be particularly helpful when treatment benefits differ significantly between patients [1]. They can be used in chronic conditions which may require long-term treatment but with insufficient evidence to support the routine use of one treatment option over another. They are best suited for treatments with a relatively rapid onset and offset of action and when objective assessments of treatment effect are possible. Given the current emphasis on patient-centered and personalized care, n-of-1 trials could be an important decision-support tool when applied in clinical practice by informing treatment decisions based on patient-derived data. Advocates of this underutilized treatment approach suggest that important outcomes may be improved when treatment decisions are individualized using n-of-1 trials, as ineffective therapies can be discontinued altogether or replaced with superior alternatives.

First described in clinical medicine by Guyatt et al. in 1986 in a patient with severe asthma [2], n-of-1 trials have not been widely adopted in clinical practice. The broad uptake of n-of-1 trials in clinical practice may be limited by a lack of evidence that their use improves outcomes compared to usual care. Numerous case reports and case series describe the application of n-of-1 trials in various populations, including attention-deficit hyperactivity disorder and other neuropsychiatric conditions, pulmonary disease, and musculoskeletal disease, including osteoarthritis [3,4]. If the n-of-1 trial approach itself is considered an intervention, it should be scrutinized with the same rigor as any other intervention in clinical practice. A randomized clinical trial comparing n-of-1 trials (intervention) against usual clinical care (control) is optimal to evaluate their effectiveness in improving patient outcomes. The objective of this review was to map the body of literature describing randomized trials comparing n-of-1 trials to routine care in any clinical field. A scoping review was performed, given the heterogeneous nature of the literature review.

## Methods

### Overview

A comprehensive literature search was performed in accordance with the Preferred Reporting Items for Systematic Reviews and Meta-Analyses guidelines [5]. The protocol was previously published and registered on PROSPERO (CRD42020166490) [6].

### Search strategy and selection criteria

Our goal was to include all prospective, parallel-group, randomized clinical trials (**RCT**s) in human subjects that assessed clinical outcomes in a group undergoing an n-of-1 trial compared to a group receiving another treatment approach. An inherent obstacle to this literature search was inconsistent nomenclature. Therefore, studies were included even if they did not specifically refer to an “n-of-1 trial”, provided the intervention met the following criteria: 1) randomized treatment periods comparing interventions tested within blocks or pairs, 2) prospective crossover of interventions, 3) single patients as the unit of analysis. We restricted our review to studies in human subjects with any medical diagnosis, treatment, or outcome, and we had no restrictions on language or date. We excluded studies that did not include a comparison group.

We searched MEDLINE (PubMed), Embase, Cochrane Library, CINAHL, PsycINFO, Scopus, and Web of Science from their inception first in October 2019 with no data limiters or filters. The searches were rerun in December 2021 and updated to address changes in database algorithms. Search terms included *n-of-1*, *personalized trial*, *single-case experimental design*, and *single-subject trial*. We searched the gray literature using the ProQuest Dissertations & Theses database and identified unpublished or ongoing studies by searching ClinicalTrials.gov and the WHO International Clinical Trials Registry Platform. The reference lists of all included studies were hand-searched. The detailed reproducible search strategies used for PubMed and Embase are provided in the Supporting Information, S1 File.

After duplicates were removed, two investigators (J.P.S. and S.H.W.) independently screened all records by title and abstract using Rayyan, a web-based application allowing blinded independent assessments [7]. Full texts were then retrieved and independently reviewed by the same two investigators to determine the final list of included studies. At both the title/abstract screening step and the full-text review step, any disagreements were resolved with discussion and arbitrated by a third investigator (D.M.) if needed. The study selection process was recorded in a Preferred Reporting Item for Systematic Reviews and Meta-Analyses (**PRISMA**) flow diagram. Pre-defined data items were extracted from the final included studies using a standardized data extraction form.

### Data analysis

Two assessments of risk of bias were performed separately for each included study. First, each randomized trial was assessed using the domain-based Cochrane Collaboration tool [8]. Next, the n-of-1 trial tested within each RCT was assessed using selected elements from the Consolidated Standards of Reporting Trials (**CONSORT**) extension for n-of-1 trials (**CENT**) checklist [9].

### Ethics

According to institutional guidelines, neither approval from the ethics committee nor informed consent from the study populations is required for literature reviews of publicly available data.

## Results

**Fig 1:**
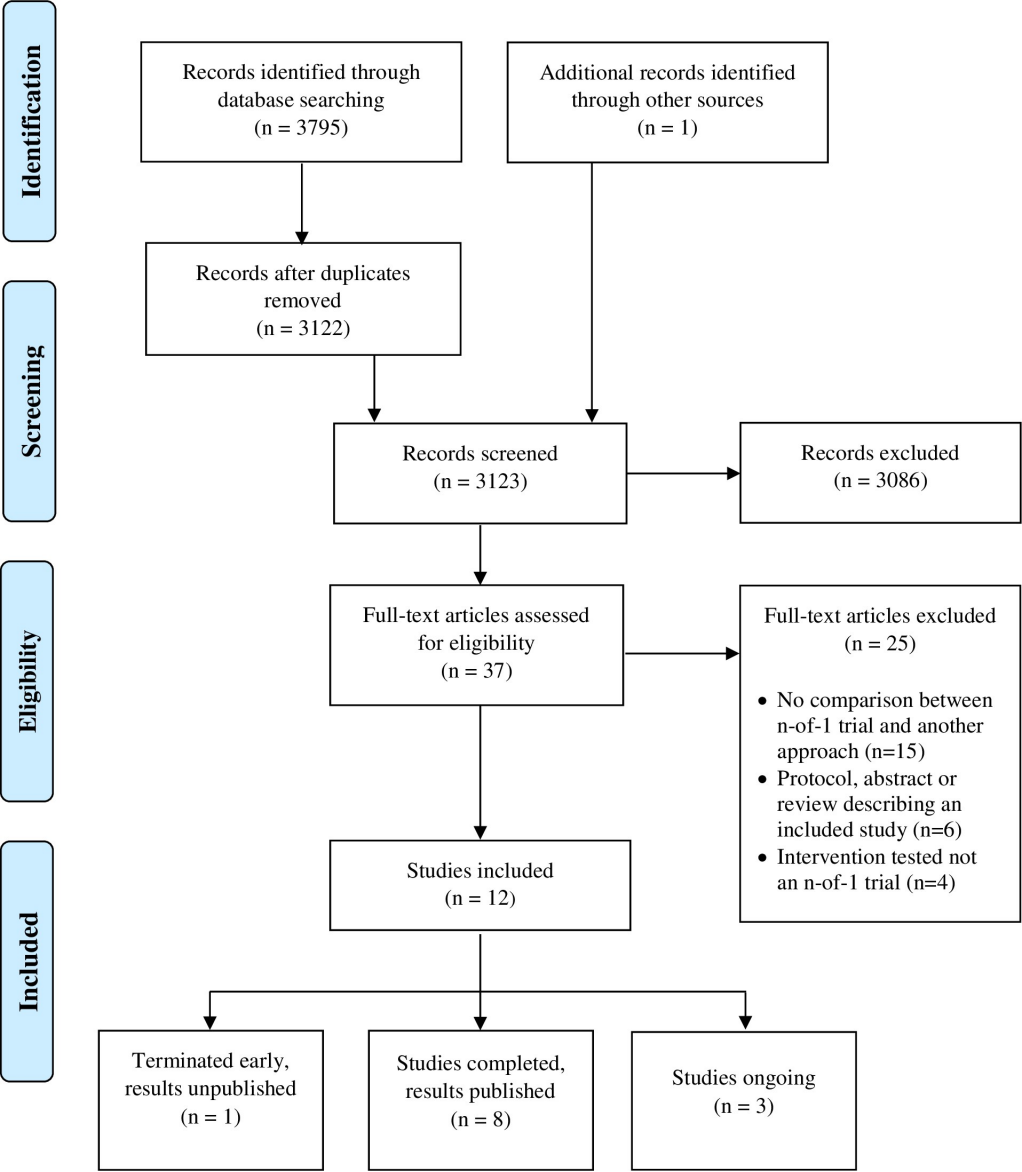
Preferred Reporting Item for Systematic Reviews and Meta-Analyses (PRISMA) flow diagram.

The database searches yielded 3122 unique records that were reviewed for eligibility criteria. After excluding 3086 records based on title and abstract, another 25 articles were excluded following full-text review (complete list of excluded full-text articles included in the Supporting Information, S2 File). One additional article [10] was identified after a review of reference lists of included articles, resulting in 12 studies selected for inclusion in this scoping review. See Fig 1 for the study selection process with reasons for exclusion. Among the 12 studies, eight were completed with published results, three were either ongoing or completed with results not published yet, and one was terminated after six patients were enrolled without results published.

Five of the trials were conducted in Canada, three in the United States, and the remaining in Israel, England, and Switzerland. Half of the trials were prospectively registered on ClinicalTrials.gov, the ISRCTN trial registry, or the WHO International Clinical Trials Registry Platform. All of the trials required informed consent; most described the use of a signed written consent while two specified a verbal consent process [10,11]. The sample sizes were relatively small, with most trials enrolling fewer than 70 patients. None of the trials reached its reported sample size target for primary outcome assessment. All of the trials were conducted in the outpatient setting and tested n-of-1 trials against what was described as usual or standard care. See Supporting Information S1 Table for a detailed summary of the trial designs.

Trials with results published

- Two randomized trials compared patient-reported pain intensity or cost-effectiveness in chronic pain management [12–14]. Two of the articles reported different outcomes from the same trial, the Personalized Research for Monitoring Pain Treatment (**PREEMPT**) study [12,13].
- Two randomized trials by the same author compared quality of life, exercise capacity, and theophylline use in adults with irreversible chronic airflow limitation [15,16]. The first trial reported six-month outcomes, and the second trial expanded the inclusion criteria and extended follow-up to 12 months.
- Two trials, one of which was quasi-randomized, compared parents’ attitudes towards using methylphenidate in treating children with attention deficit hyperactivity disorder [10,17].
- One randomized trial compared quality of life and number of atrial fibrillation events in adults with symptomatic atrial fibrillation [18].

Trials without results published

- A completed randomized trial compared blood pressure reduction, side effect experience, and patient satisfaction among children with hypertension. The results are not published yet [11].
- An ongoing randomized trial is comparing adherence to statin therapy among adults who have previously discontinued or refused statin therapy [21].
- An ongoing randomized trial is comparing the number of pain medications prescribed and patient-reported pain intensity in chronic pain management [19].
- One trial was terminated after one year with six patients enrolled (sample size target 30) and planned to compare cholesterol levels and the number of patients taking statin medications among diabetic adults with an indication for a statin [20].

**Fig 2:**
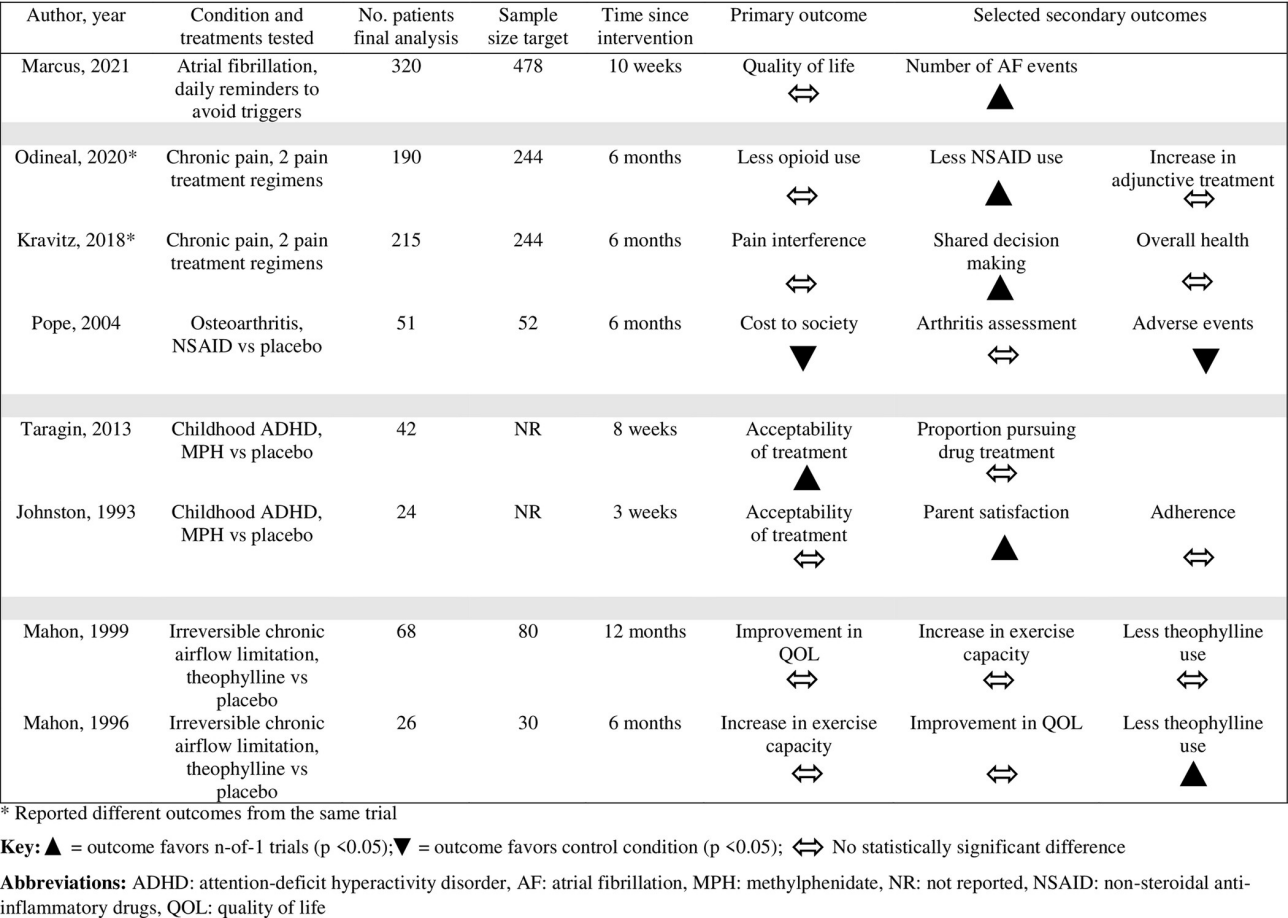
Summary of primary outcome and selected secondary outcomes from studies with results published.

It was not possible to quantitatively combine the results of the studies due to clinical heterogeneity in the populations, diseases, treatments, and outcome measures. See Fig 2 for a summary of trial results.

Only one of the studies showed benefit of n-of-1 trials in the pre-specified primary outcome [17]. This trial, which was not truly randomized, showed higher acceptability of methylphenidate treatment among parents whose children underwent an n-of-1 trial compared to those who were treated according to usual care. Five studies identified statistically significant results favoring the use of n-of-1 trials in at least one of the secondary outcomes, including fewer atrial fibrillation events [20], decreased medication use [13,15], increased medication-related shared decision making [12], and increased parent satisfaction with care [10].

The domain-based Cochrane Collaboration tool was used to assess the risk of bias for the eight trials with results published [8]. This tool was developed by the Cochrane Collaboration to cover six domains of bias: selection bias, performance bias, detection bias, attrition bias, reporting bias, and other bias. Selected items from these domains are presented in Table 1.

**Table 1:** Risk of bias of randomized trials.

	Random sequence generation	Allocation concealment	Blinding: participants and personnel	Blinding: outcome assessment	Incomplete outcome data	Selective reporting
Marcus 2021[18]	+	+	-	-	-	+
Odineal 2020[13] Kravitz 2018[12]	+	+	-	?	+	+
				-	+	+
Taragin 2013[17]	-	-	-	-	-	+
Pope 2004[14]	?	?	-	+	+	+
Mahon 1999[16]	+	+	-	+	+	+
Mahon 1996[15]	-	-	-	+	+	+
Johnston 1993[10]	-	-	-	+	+	+
Tudor 2020[21]	Unable to assess, results not published.					
Buclin 2018[19]						
Samuel 2018[11]						
McDonald 2008[20]						

Assess as low (+), unclear (?), or high (-) risk of bias present.

In two trials, the allocation process was at high risk for bias. In Taragin 2013, participants were not randomized to treatment groups [17]. Patients were referred by their pediatrician to one of three consulting clinics, one of which routinely employed n-of-1 trials while the other clinics offered usual care. Outcomes across the clinics were then compared. In Mahon 1996, participants were randomized with a coin toss by a person unaware of the participant’s baseline characteristics [15].

Blinding the participants to treatment assignment (n-of-1 trial versus control condition) is not feasible given the patient-centered nature of n-of-1 trials, and none of the trials attempted to do so. Four trials reported that the outcome assessor was blinded to treatment assignments [10,14–16]. The primary outcome was patient-reported in three studies, and patients were not blinded to treatment groups [12,17]. In one study the primary outcome was derived from the medical record, and it was not clear whether the outcome assessors were blinded to treatment groups [13].

Studies were considered at high risk for attrition bias if more than 20% of participants were excluded from the analysis of the primary outcome. Five studies provided outcome data for at least 80% of participants [10,12–16]. One study was considered at high risk for attrition bias as eight-week outcome data was provided for fewer than 50% of participants [17].

The CONSORT (Consolidated Standards of Reporting Trials) extension for N-of-1 trials (CENT 2015) provides recommendations to promote standardized reporting of n-of-1 trials [9]. In Table 2, some elements of the CENT 2015 checklist were adapted to assess for risk of bias in the designs of the n-of-1 trials tested within each RCT.

**Table 2:** Risk of bias of n-of-1 trial design.

	Trial design	Randomized treatment order	Blinding of participants and personnel	Interventions described in detail	Pre-specified outcomes
Marcus 2021[18]	+	+	-	+	+
Odineal 2020[13] Kravitz 2018[12]	+	+	-	+	+
Taragin 2013[17]	+	+	+	+	-
Pope 2004[14]	+	+	+	+	+
Mahon 1999[16]	+	+	+	+	+
Mahon 1996[15]	+	+	+	+	+
Johnston 1993[10]	-	-	+	+	+

Assess as low (+), unclear (?), or high (-) risk of bias.

Most of the trials reported the n-of-1 trial protocol design with sufficient detail, however one trial did not describe the planned number of treatment periods or duration of each period [10].

Most of the trials described randomization of the order of treatment periods and the methods used to generate the allocation sequence. One trial did not describe randomization of treatment order or the intended sequence of periods [10].

Most of the trials reported that patients, families, and personnel were blinded to the treatment assignment and used identical placebos [10,14–17]. The PREEMPT study was open-label. The treatments being tested were individualized for each participant and could not reasonably be masked (ex. pharmacological therapy may have been tested against complementary therapies such as massage or meditation) [12,13].

Most of the trials provided completely defined pre-specified outcome measures, including a description of the measurement properties of the outcome assessment tools and a pre-specified protocol for determining the result of the n-of-1 trial [10,12–16]. One study did not describe how often the treatment effect was assessed within the n-of-1 trial nor the criteria to adjudicate the result of the n-of-1 trial [17].

## Discussion

We comprehensively reviewed the body of literature describing randomized trials testing the n-of-1 approach. The studies encompassed only six broad disease categories: atrial fibrillation, chronic pain, chronic airflow limitation, attention-deficit hyperactivity disorder, pediatric hypertension, and perceived statin intolerance. N-of-1 trials could potentially be helpful in various diseases, as long as the baseline condition is stable and chronic, the treatments have a relatively rapid onset and offset of action, and objective measurements of treatment success or failure are possible. Additional disease states amenable to n-of-1 trials include diabetes, irritable bowel syndrome, depression, asthma, and insomnia.

Inconsistency in terminology was a barrier to systematically searching the literature for randomized trials testing the n-of-1 trial approach. Among the full-text articles we reviewed, an assortment of synonyms was used, including *personalized trials*, *medication trials*, *single-subject trials*, *within-patient controlled trials*, *single-patient multi-crossover trials*, *n-of-1 studies*, *n-of-1 experiments*, and *single-case experimental design*. CENT guidelines advise against using heterogeneous terminology and instead recommend using “n-of-1 trial” in the title to optimize the identification of relevant studies in electronic database searches [9]. The terms mentioned above are especially problematic when they contain words that are pervasive in the medical literature (ex. *medication trial*), making search queries designed to look for these terms result in numerous irrelevant articles for screening. The National Library of Medicine uses medical Subject Headings (MeSH) to organize vocabulary used in indexing and searching MEDLINE/PubMed and other NLM databases. At our initial literature search in 2019, no relevant MeSH terms existed to describe n-of-1 trials. In 2020 a new MeSH term was released to include n-of-1 trials (*single-case studies*, MeSH Unique ID: D000080907), and we incorporated this term in our updated literature search in 2021. The creation of this MeSH term may improve future queries of the medical literature for n-of-1 trials.

Most of the trials were small single-center studies (sample sizes ranged from 24 to 320 participants) with limited power to identify small yet clinically meaningful differences. Some studies did not specify a sample size calculation [10,17], and one trial was terminated after one year due to insufficient enrollment [20]. The largest trial to date, the I-STOP-AFib trial [18], was a fully remote mobile application-based trial that recruited 446 participants using email invitations to participants of an international web-based cardiovascular cohort study (the Health eHeart Study) and members of an patient advocacy organization. While this approach facilitated a high recruitment rate, the attrition rate was nearly 30%, a common problem with remote-only randomized studies [22]. The second-largest trial to date, the PREEMPT study [12,13], recruited 215 patients with chronic musculoskeletal pain. Factors that likely increased enrollment rates included studying a common condition, allowing flexibility in the treatments to be tested, remote data collection, and recruitment from multiple clinics across a broad geographical region. The customizable nature of the n-of-1 trial design, with treatments and treatment length chosen according to clinician judgment and patient preferences, may have increased willingness to participate. Identifying the facilitators and barriers to recruitment and enrollment in these trials will improve the feasibility of adequately powered trials in the future.

While most of the study results either favored the use of n-of-1 trials or had inconclusive results, one study showed potentially negative effects of n-of-1 trials [14]. This study sought to assess the cost-effectiveness of n-of-1 trials in identifying whether to prescribe non-steroidal anti-inflammatory drugs to patients with osteoarthritis. In an attempt to balance the potential bias of frequent visits and outcome measurements in the n-of-1 trial group, the control group was offered more frequent visits than in usual clinical practice. Both groups were seen at the same intervals, but the costs of the extra visits were not included in the control patients. This may have resulted in both groups experiencing the benefit of increased visits, but with the costs only applied to the n-of-1 group. In addition, although more drug-related side effects were reported in the n-of-1 trial group, the authors suggest that the n-of-1 trial patients were asked more meticulously about side effects, which may have led to overreporting. Although there were no significant differences between groups in this small study (n = 51), measures of pain and disability improved more in the n-of-1 trial group than the standard treatment group.

Technological advances may mitigate some of the costs and inconveniences of n-of-1 trials as remote patient monitoring can reduce the need for repeated in-person visits. The I-STOP-AFib and the PREEMPT studies used a mobile device-supported application to design the customized n-of-1 trial, randomize the treatment sequence, notify patients of treatment changes, collect patient-reported outcome data, and display the final results for clinician or patient use. The I-STOP-AFib study also used a mobile electrocardiogram recording device paired with smartphones to allow remote recording of cardiac rhythms when the patient experienced a sensation of an atrial fibrillation episode. As remote monitoring of clinical markers and side effects becomes more convenient and less expensive, the cost-effectiveness and ease of conducting n-of-1 trials are likely to improve.

## Conclusions

Insufficient sample sizes likely precluded these studies from identifying statistically significant results using frequentist analyses. The question of whether the n-of-1 trial approach will improve clinically meaningful outcomes if applied to a large swath of patients remains unanswered. N-of-1 trials could also be preferred if they result in equivalent clinical outcomes but greater patient satisfaction and fewer office visits. Adequately powered trials are needed to understand their potential role in improving patient satisfaction, adherence, and clinical outcomes. We may find that just as patients show differences in their responses to treatment options, they may also vary in whether n-of-1 trials can improve their outcomes.

## Supporting information

S1 File Detailed search strategy. (DOCX)

S2 File List of studies excluded at full-text screening stage. (DOCX)

S1 Table Characteristics of twelve included studies. (DOCX)
