# Supplementary material for: A scoping review of randomized trials assessing the impact of n-of-1 trials on clinical outcomes
Source: PLoS One. 2022 Jun 2;17(6):e0269387. doi: 10.1371/journal.pone.0269387 (PMC9162303; doi:10.1371/journal.pone.0269387)
Supplement: S1 File — (DOCX) [file pone.0269387.s001.docx]

**S1 File: Detailed Search Strategy**

**PubMed**

Original search (October 22, 2019):

((("n-of-1" OR "n of 1" OR "n-of-one" OR precision medicine trial* OR single patient trial* OR single subject trial* OR personalized medicine trial* OR personalised medicine trial*))) OR ((((single subject design* OR single case design* OR self-experiment*))) AND ((trial*[tiab] OR trial*[ot])))

Update search (December 24, 2021):

"Single-Case Studies as Topic"[Mesh] OR "n-of-1" OR "n of 1" OR "n-of-one" OR "precision medicine trial*" OR "single patient trial*" OR "single subject trial*" OR "personalized medicine trial*" OR (("personalised medicine" OR "single subject design*" OR "single case design*" OR "self-experiment*") AND ("trial*"[tiab] OR "trial*"[ot]))
Filters: 10/2019-3000/12/12

**Embase**

Original search (October 22, 2019):

#1. 'n-of-1' OR 'n of 1' OR 'n-of-one' OR 'single subject trial*' OR 'personali?ed medicine trial*' OR 'precision medicine trial*' OR 'personali?ed trial*' OR 'single patient trial*' OR 'single person trial*'

#2. trial*:ab,kw,ti

#3. 'single case design*' OR 'self-experiment' OR 'single subject design*'

#4. #2 AND #3

#5. #1 OR #4

Update search (December 24, 2021):

#1 - 'n-of-1' OR 'n of 1' OR 'n-of-one' OR 'single subject trial*' OR 'personali?ed medicine trial*' OR 'precision medicine trial*' OR 'personali?ed trial*' OR 'single patient trial*' OR 'single person trial*

#2 - trial*:ab,kw,ti

#3 - 'single case design*' OR 'self-experiment' OR 'single subject design*'

#4 - #2 AND #3

#5 - #1 OR #4

Publication years: 2019-2022

**Other database search dates**

ProQuest Dissertations & Theses - 10/10/2019, unable to update search in 2021 due to lack of institutional access

Web of Science - 10/10/2019, 12/24/2021

CINAHL - 10/22/2019, 12/24/2021

ClinicalTrials.gov - 10/22/2019, 12/23/2021

Cochrane - 10/22/2019, 12/24/2021

PsycInfo - 10/22/2019, 12/24/2021

Scopus - 10/22/2019, unable to update search in 2021 due to lack of institutional access

WHO International Clinical Trials Database - 10/25/2019. 12/23/2021
