# Supplementary material for: A scoping review of randomized trials assessing the impact of n-of-1 trials on clinical outcomes
Source: PLoS One. 2022 Jun 2;17(6):e0269387. doi: 10.1371/journal.pone.0269387 (PMC9162303; doi:10.1371/journal.pone.0269387)
Supplement: S2 File — (DOCX) [file pone.0269387.s002.docx]

**S2 File: List of studies excluded at full-text screening stage**

Twenty-five articles were excluded after full-text review.

- Fifteen articles were excluded because there was no comparison between n-of-1 trials and another treatment strategy
- Six articles were excluded because they were separately published protocol, abstract, or narrative review describing an included study.
- Four articles did not use an n-of-1 trial.

Fifteen articles were excluded because there was no comparison between n-of-1 trials and another treatment strategy

1. Melatonin in Youth: N-of-1 Trials in a Stimulant-treated Attention Deficit Hyperactivity Disorder (ADHD) Population. ClinicalTrials.gov. NCT02333149. https://clinicaltrials.gov/show/NCT02333149
2. Massive Individualized N-of-1 Experiments (MINEs) (MINEs). ClinicalTrials.gov. NCT03695263. https://clinicaltrials.gov/ct2/show/NCT03695263
3. Johannessen T, Kristensen P, Petersen H, Fosstvedt D, Løge I, Kleveland PM, Dybdahl J. The symptomatic effect of 1-day treatment periods with cimetidine in dyspepsia. Combined results from randomized, controlled, single-subject trials. *Scand J Gastroenterol.* 1991 Sep;26(9):974-80.
4. Freeston, M. H., Ladouceur, R., Rhéaume, J., & Léger, E. (1998). Applications of cognitive models of OCD in clinical practice. In E. Sanavio (Ed.), Behavior and cognitive therapy today: Essays in honor of Hans J. Eysenck (pp. 117–126). Elsevier Science Ltd.

https://doi.org/10.1016/B978-008043437-7/50010-7

1. Price JD, Grimley Evans J. N-of-1 randomized controlled trials ('N-of-1 trials'): singularly useful in geriatric medicine. *Age Ageing.* 2002 Jul;31(4):227-32.
2. Avins AL, Bent S, Neuhaus JM. Use of an embedded N-of-1 trial to improve adherence and increase information from a clinical study. *Contemp Clin Trials*. 2005 Jun;26(3):397-401. doi: 10.1016/j.cct.2005.02.004. Epub 2005 Mar 28. Erratum in: Contemp Clin Trials. 2006 Feb;27(1):101-2.
3. Brossart, Daniel & Meythaler, Jay & Parker, Richard & McNamara, James & Elliott, Timothy. (2008). Advanced Regression Methods for Single-Case Designs: Studying Propranolol in the Treatment for Agitation Associated With Traumatic Brain Injury. *Rehabilitation Psychology.* 53. 357-369.
4. Stunnenberg BC, Woertman W, Raaphorst J, Statland JM, Griggs RC, Timmermans J, Saris CG, Schouwenberg BJ, Groenewoud HM, Stegeman DF, van Engelen BG, Drost G, van der Wilt GJ. Combined N-of-1 trials to investigate mexiletine in non-dystrophic myotonia using a Bayesian approach; study rationale and protocol. *BMC Neurol*. 2015 Mar 25;15:43.
5. Punja S, Schmid CH, Hartling L, Urichuk L, Nikles CJ, Vohra S. To meta-analyze or not to meta-analyze? A combined meta-analysis of N-of-1 trial data with RCT data on amphetamines and methylphenidate for pediatric ADHD. *J Clin Epidemiol*. 2016 Aug;76:76-81.
6. Punja S, Nikles CJ, Senior H, Mitchell G, Schmid CH, Heussler H, Witmans M, Vohra S. Melatonin in Youth: N-of-1 trials in a stimulant-treated ADHD Population (MYNAP): study protocol for a randomized controlled trial. *Trials*. 2016 Jul 29;17:375.
7. Cutler AJ, Oliveira J, Ferreira RC, Challis B, Walker NM, Caddy S, Lu J, Stevens HE, Smyth DJ, Pekalski ML, Kennet J, Hunter KMD, Goodfellow I, Wicker LS, Todd JA, Waldron-Lynch F. Capturing the systemic immune signature of a norovirus infection: an n-of-1 case study within a clinical trial. *Wellcome Open Res*. 2017 Oct 5;2:28.
8. Stunnenberg BC, Raaphorst J, Groenewoud HM, Statland JM, Griggs RC, Woertman W, Stegeman DF, Timmermans J, Trivedi J, Matthews E, Saris CGJ, Schouwenberg BJ, Drost G, van Engelen BGM, van der Wilt GJ. Effect of Mexiletine on Muscle Stiffness in Patients With Nondystrophic Myotonia Evaluated Using Aggregated N-of-1 Trials. *JAMA*. 2018 Dec 11;320(22):2344-2353.
9. Stunnenberg B., Raaphorst J., Groenewoud H., Statland J., Griggs R., Woertman W., et al. A series of aggregated randomized-controlled N-of-1 trials with mexiletine in non-dystrophic myotonia: clinical trial results and validation of rare disease design (P3.440) *Neurology* Apr 2018, 90 (15 Supplement) P3.440
10. Bech AP, Wetzels JFM, Groenewoud H, Nijenhuis T. The Use of N-of-1 Trials to Individualize Treatment in Patients With Renal Magnesium Wasting. *Am J Kidney Dis*. 2019 Feb;73(2):288-290. doi: 10.1053/j.ajkd.2018.08.010. Epub 2018 Oct 11. PMID: 30318133.
11. Edelson J, Byrnes J, Mitchell G, Heussler H, Melaku M, Nikles J. Protocol for a longitudinal study of melatonin therapy and cost effectiveness analysis in stimulant-treated children with ADHD and insomnia: An N-of-1 trial. *Contemp Clin Trials Commun*. 2020 Jan 22;17:100530. doi: 10.1016/j.conctc.2020.100530. PMID: 32025588; PMCID: PMC6997492.

Six articles were excluded because they were separately published protocol, abstract, or narrative review describing an included study.

1. Mahon, J, Laupacis, A, Donner, A, Wood, T. A randomized trial of n-of-1 trials versus conventional therapy. Clinical Research. 41 (2) A180, (abstract). April 1993.  **[abstract only of study described in Mahon 1996]**
2. Correction: Randomized study of n of 1 trials versus standard practice (BMJ) 27th April (1069-1074). BMJ. **[correction of study described in Mahon 1996]**
3. Chatellier G. Une étude randomisée comparant les résultants d'essais de taille 1 à la méthode habituelle de distribution de soins [Randomized study of n-of-1 trials versus standard practice]. Rev Epidemiol Sante Publique. 1996 Aug;44(4):382-3. French. **[review paper providing overview of study described in Mahon 1996]**
4. Pope J, Stevens A, Fenlon D, Mahon, J. A randomized trial of conventional treatment versus n of 1 trials of NSAIDs in osteoarthritis (OA). Cost effectiveness of treatment (Rx) strategies. Arthritis & Rheumatism. 41(9) (Supplement):S79, (abstract) September 1998. **[abstract only of study described in Pope 2004]**
5. Can within-patient controlled trials help to personalize the treatment of chronic pain? Cochrane Central Register of Controlled Trials. Added to CENTRAL: 31 July 2019. ISRCTN45725581. http://www.who.int/trialsearch/Trial2.aspx?TrialID=ISRCTN45725581 **[duplicate registry of study protocol described in Buclin 2018]**
6. Barr C, Marois M, Sim I, Schmid CH, Wilsey B, Ward D, Duan N, Hays RD, Selsky J, Servadio J, Schwartz M, Dsouza C, Dhammi N, Holt Z, Baquero V, MacDonald S, Jerant A, Sprinkle R, Kravitz RL. The PREEMPT study - evaluating smartphone-assisted n-of-1 trials in patients with chronic pain: study protocol for a randomized controlled trial. Trials. 2015 Feb 27;16:67. **[protocol published for study described in Kravitz 2018]**

Four articles did not use an n-of-1 trial.

1. Vijverberg SJ, Pijnenburg MW, Hövels AM, Koppelman GH, Maitland-van der Zee AH. The need for precision medicine clinical trials in childhood asthma: rationale and design of the PUFFIN trial. *Pharmacogenomics*. 2017 Mar;18(4):393-401.
2. Cattivelli R, Castelnuovo G, Musetti A, Varallo G, Spatola CAM, Riboni FV, Usubini AG, Tosolin F, Manzoni GM, Capodaglio P, Rossi A, Pietrabissa G, Molinari E. ACTonHEALTH study protocol: promoting psychological flexibility with activity tracker and mHealth tools to foster healthful lifestyle for obesity and other chronic health conditions. *Trials*. 2018 Nov 29;19(1):659.
3. Browning M, Bilderbeck AC, Dias R, Dourish CT, Kingslake J, Deckert J, Goodwin GM, Gorwood P, Guo B, Harmer CJ, Morriss R, Reif A, Ruhe HG, van Schaik A, Simon J, Sola VP, Veltman DJ, Elices M, Lever AG, Menke A, Scanferla E, Stäblein M, Dawson GR. The clinical effectiveness of using a predictive algorithm to guide antidepressant treatment in primary care (PReDicT): an open-label, randomised controlled trial. *Neuropsychopharmacology*. 2021 Jun;46(7):1307-1314. doi: 10.1038/s41386-021-00981-z. Epub 2021 Feb 26. PMID: 33637837; PMCID: PMC8134561.
4. García-Estela A, Angarita-Osorio N, Alonso S, Polo M, Roldán-Berengué M, Messaggi-Sartor M, Mur-Mila E, Vargas-Puertolas L, Pérez V, Duarte E, Colom F. Improving Depressive Symptoms through Personalised Exercise and Activation (IDEA): Study Protocol for a Randomised Controlled Trial. *Int J Environ Res Public Health*. 2021 Jun 10;18(12):6306. doi: 10.3390/ijerph18126306. PMID: 34200805; PMCID: PMC8296117.
