# Supplementary material for: A scoping review of randomized trials assessing the impact of n-of-1 trials on clinical outcomes
Source: PLoS One. 2022 Jun 2;17(6):e0269387. doi: 10.1371/journal.pone.0269387 (PMC9162303; doi:10.1371/journal.pone.0269387)
Supplement: S1 Table — (DOCX) [file pone.0269387.s003.docx]

**S1 Table: Characteristics of twelve included studies** (ordered by year)

| **Marcus 2021** | |
| --- | --- |
| **Methods** | **Study design:** parallel group RCT  **Follow-up period:** 10 weeks  **Funding:** Patient-Centered Outcomes Research Institute and National Institutes of Health (National Institute of Biomedical Imaging and Bioengineering)  **Hypothesis:** Participation in an n-of-1 trial of self-selected atrial fibrillation (AF) triggers would enhance AF-related quality of life |
| **Participants** | **Setting:** remote mobile-application based intervention, participants primarily recruited through email subscribers of the Health eHeart Study and StopAfib.org  **Country:** United States  **Population:** adults with symptomatic AF |
| **Interventions** | **N-of-1 trial:** Trigger testing using n-of- trial, comparing six 1-week periods of daily text messages reminding participant to either avoid or expose themselves to potential AF trigger (trigger selected by participant). Six periods randomly assigned in 3 blocks of trigger on/trigger off or trigger off/trigger on. Daily queries regarding trigger exposure in preceding day and presence of AF episodes  **Control condition:** Usual care, and daily queries regarding the presence of AF episodes |
| **Outcomes** | **Primary outcome:** Improvement in AF-related quality of life, measured by validated questionnaire, Atrial Fibrillation Effect on Quality-of-Life (AFEQT) from baseline to the end of the initial 10-week study period  **Secondary outcome:** number of AF events during the final 4 weeks of the initial study period |
| **Trial status** | Published.  Marcus GM, Modrow MF, Schmid CH, Sigona K, Nah G, Yang J, Chu TC, Joyce S, Gettabecha S, Ogomori K, Yang V, Butcher X, Hills MT, McCall D, Sciarappa K, Sim I, Pletcher MJ, Olgin JE. Individualized Studies of Triggers of Paroxysmal Atrial Fibrillation: The I-STOP-AFib Randomized Clinical Trial. JAMA Cardiol. 2021 Nov 14:e215010. doi: 10.1001/jamacardio.2021.5010. Epub ahead of print. |
|  |  |
| **Tudor 2020** | |
| **Methods** | **Study design:** feasibility study, three-arm parallel group RCT to inform the design of a future trial  **Follow-up period:** 6 months  **Funding:** National Institute of Health Research (NIHR) Collaboration for Leadership in Applied Health Research and Care, Oxford; NIHR Oxford Biomedical Research Centre  **Hypothesis:** n-of-1 trial endorsed and delivered by a general practitioner will increase adherence to statin therapy compared to usual care |
| **Participants** | **Setting:** primary care clinics  **Country:** England  **Population:** adults who have previously discontinued or refused statin treatment following a recommendation from a clinician |
| **Interventions** | **Unblinded n-of-1 trial (intervention):** comparing open label treatment with statin versus no treatment. Three paired treatment periods with a prespecified treatment order (no treatment (A) followed by statin (B), ABABAB for all participants, with 4 weeks per treatment period. Online questionnaire data collection every day for the last 7 days of each 4-week treatment period regarding adherence, symptoms, attributions, and pain  **Blinded n-of-1 trial (positive control):** comparing blinded treatment with statin versus placebo. Three paired treatment periods with prespecified treatment order for first treatment pair (AB), and randomized order within pairs for 2^nd^ and 3^rd^ treatment pairs. Online questionnaire data collection every day for the last 7 days of each 4-week treatment period regarding adherence, symptoms, attributions, and pain  **Control condition:** Usual care |
| **Outcomes** | **Primary outcome:** feasibility of an n-of-1 trial to test adverse events and designed to increase adherence to statin therapy as assessed by 1) proportion of invited patients who enroll, 2) proportion of enrolled participants who are randomized to n-of-1 trial who agree to engage in n-of-1 trial, 3) proportion in each arm who decide to continue statin therapy |
| **Trial status** | Recruitment completed per International Clinical Trials Registry Platform ISRCTN11142694, last updated 11/08/2021 |
|  |  |
| **Kravitz 2018 and Odineal 2020** | |
| **Methods** | **Study design:** parallel group RCT  **Follow-up period:** 6 months  **Funding:** National Institutes of Health (National Institute of Nursing Research and the National Center for Advancing Translational Sciences)  **Hypothesis:** Participation in an n-of-1 trial improves pain-related or patient-engagement outcomes compared with usual care |
| **Participants** | **Setting:** primary care clinics  **Country:** United States  **Population:** adults with chronic musculoskeletal pain |
| **Interventions** | **N-of-1 trial:** comparing open label treatment with 2 pain regimens. Regimens made up of one or more treatments selected by patient and clinician from 8 treatment categories. Minimum two paired treatment periods (each treatment tested at least 2 times), random order, 1-2 weeks per treatment period. Comparison using daily questionnaires assessing pain intensity, pain interference, and side effects.  **Control condition:** Usual care |
| **Outcomes** | **Kravitz 2018**  **Primary outcome:** change in Patient-Reported Outcomes Measurement Information System (PROMIS) pain-related interference 8-item short-form scale  **Secondary outcomes:** pain intensity, physical and mental global health, analgesic adherence, patient-clinician relationship, and satisfaction with pain care |
|  | **Odineal 2020**  **Primary outcome:** changes in analgesic prescription by medical record review |
| **Trial status** | Published.  Kravitz RL, Schmid CH, Marois M, Wilsey B, Ward D, Hays RD, Duan N, Wang Y, MacDonald S, Jerant A, Servadio JL, Haddad D, Sim I. Effect of Mobile Device-Supported Single-Patient Multi-crossover Trials on Treatment of Chronic Musculoskeletal Pain: A Randomized Clinical Trial. JAMA Intern Med. 2018 Oct 1;178(10):1368-1377.  Odineal DD, Marois MT, Ward D, Schmid CH, Cabrera R, Sim I, Wang Y, Wilsey B, Duan N, Henry SG, Kravitz RL. Effect of Mobile Device-Assisted N-of-1 Trial Participation on Analgesic Prescribing for Chronic Pain: Randomized Controlled Trial. J Gen Intern Med. 2020 Jan;35(1):102-111. |
|  |  |
| **Buclin 2018** | |
| **Methods** | **Study design:** parallel group RCT  **Follow-up period:** 6 months  **Funding:** Swiss National Science Foundation (private sector organization)  **Hypothesis:** n-of-1 trials are more successful in detecting medication inefficacy or efficacy than standard practice (definition of success: deprescription or decrease of pain by at least 30%, respectively) |
| **Participants** | **Setting:** pain centers  **Country:** Switzerland  **Population:** adults with pain of any origin lasting at least 3 months and patient or practitioner has doubts about the efficacy of a given medication |
| **Interventions** | **N-of-1 trial:** double-blinded comparison of one medication (chosen by patient or practitioner) with placebo. Three paired 2-week treatment periods (each treatment tested 3 times), random order. Comparison using daily pain and adverse effects evaluation by patient.  **Control condition:** standard management of chronic pain currently offered in pain centers |
| **Outcomes** | **Primary outcome:** Number of pain medications (medical record review) and average pain intensity (patient reported with visual analog scale)  **Secondary outcomes:** quality of life, cost estimation, consumption of analgesic medications and other pain interventions, patient’s emotional state, patient’s daily-life functioning, patient’s and physician’s reactions towards the n-of-1 approach |
| **Trial status** | Study ongoing. Currently enrolling as of ISRCTN registry: SRCTN45725581, last updated 12/31/20. |
|  |  |
| **Samuel 2018** | |
| **Methods** | **Study design:** parallel group RCT  **Follow-up period:** 6 months  **Funding:** University of Texas Health Science Center, Houston  **Hypothesis:** n-of-1 trials are superior to usual care in normalizing blood pressure while minimizing exposure to compliance-reducing side effects |
| **Participants** | **Setting:** outpatient pediatric hypertension clinic  **Country:** United States  **Population:** children and young adults (ages 10-22) with hypertension requiring pharmacologic therapy |
| **Interventions** | **N-of-1 trial:** comparing open label treatment with two active treatments (selected based on underlying condition). Minimum two paired 2-week treatment periods (each treatment tested 2 times), random order. Comparison using 24-hour ambulatory blood pressure monitoring and side effect questionnaire on final day of treatment period.  **Control condition:** usual care, treatment selected according to physician preference |
| **Outcomes** | **Primary outcome:** % of patients with well-controlled ambulatory blood pressure  **Secondary outcomes:** side effect experience, cost effectiveness, patient satisfaction |
| **Trial status** | Study completed May 2021 as of ClinicalTrials.gov registry: NCT03461003, last updated 05/04/2021. |
|  |  |
| **Taragin 2013** | |
| **Methods** | **Study design:** non-randomized observational study. Patients referred by PCP to one of three clinics. One of the clinics routinely used n-of-1 trials and the other two used usual care.  **Follow-up period:** 8 weeks  **Funding:** none reported  **Hypothesis:** parents who undergo an n-of-1 trial would be more positive about treatment with methylphenidate (MPH) |
| **Participants** | **Setting:** three referral clinics  **Country:** Israel  **Population:** Children with attention-deficit hyperactivity disorder and their parents |
| **Interventions** | **N-of-1 trial:** double blinded comparison of MPH versus identical placebo. One paired comparison with 1-week treatment periods, random order. Comparison using Conners’ Parent and Teacher Rating Scales used to assess treatment response.  **Control condition:** traditional prescription approach- MPH prescription immediately following the diagnosis and scheduled follow-up |
| **Outcomes** | **Primary outcome:** Acceptability of treatment, as measured by change in Abbreviated Acceptability Rating Profile related to drug treatment from baseline  **Secondary outcomes:** adherence to MPH treatment |
| **Trial status** | Published.  Taragin D, Berman S, Zelnik N, Karni A, Tirosh E. Parents' attitudes toward methylphenidate using n-of-1 trial: a pilot study. Atten Defic Hyperact Disord. 2013 Jun;5(2):105-9. |
|  |  |
| **McDonald 2008** | |
| **Methods** | **Study design:** parallel group RCT  **Follow-up period:**  not specified  **Funding:** sponsored by Lawson Health Research Institute  **Hypothesis:** n-of-1 trials will improve statin adherence, thereby improving low density lipoprotein cholesterol (LDL-C) levels. |
| **Participants** | **Setting:** not specified  **Country:** Canada  **Population:** adults with diabetes mellitus, indication for a statin, and willingness to retry despite previous intolerance |
| **Interventions** | **N-of-1 trial:** comparing 1-month courses of either simvastatin or placebo. Number of treatment cycles and randomization of treatment order not specified. Assessment of treatment effects not specified.  **Control condition:** standard practice, patients will be given a prescription by the doctor in the usual way |
| **Outcomes** | **Primary outcome:** mean LDL level  **Secondary outcomes:** proportion of patients taking statins at the end of the trial |
| **Trial status** | Study terminated due to insufficient recruitment/enrollment. ClinicalTrials.gov registry: NCT 00299169, last updated 1/8/2008. |
|  |  |
| **Pope 2004** | |
| **Methods** | **Study design:** parallel group RCT  **Follow-up period:** 6 months  **Funding:** Physicians Services Incorporated Foundation, study medication supplied by pharmaceutical company  **Hypothesis:** N of 1 trials may be cost-effective relative to conventional practice, with equal or better outcome measurements. |
| **Participants** | **Setting:** recruitment from outpatient rheumatology practices and newspaper advertisements  **Country:** Canada  **Population:** adults with osteoarthritis and uncertainty that NSAIDs are helpful |
| **Interventions** | **N-of-1 trial:** comparing double blinded treatment with NSAID (diclofenac) versus identical placebo. One to three pairs of 2-week treatment periods (each treatment tested 1-3 times), random order. Comparison using global assessment scores assessed via Stanford Health Assessment Questionnaire and Western Ontario and McMaster Universities Osteoarthritis Index on final day of treatment period.  **Control condition:** conventional treatment. Stop NSAID and observe response. Treatment chosen according to standard care by their own physician and the study investigator following guidelines for osteoarthritis treatment. |
| **Outcomes** | **Primary outcome:** cost-effectiveness based on society perspective  **Secondary outcomes:** treatment efficacy |
| **Trial status** | Published.  Pope JE, Prashker M, Anderson J. The efficacy and cost effectiveness of N of 1 studies with diclofenac compared to standard treatment with nonsteroidal antiinflammatory drugs in osteoarthritis. J Rheumatol. 2004 Jan;31(1):140-9. |
|  |  |
| **Mahon 1999** | |
| **Methods** | **Study design:** parallel group RCT  **Follow-up period:** 12 months  **Funding:** Ontario Ministry of Health, study medication supplied by pharmaceutical company  **Hypothesis:** patients with irreversible CAL who were given theophylline guided by n of 1 trials would have better quality of life, exercise capacity, and less theophylline use than patients treated according to standard practice. |
| **Participants** | **Setting:** four outpatient clinics in two tertiary care Canadian centers  **Country:** Canada  **Population:** adults with chronic irreversible airflow limitation both with and without history of previous theophylline use. |
| **Interventions** | **N-of-1 trial:** double blinded comparison of theophylline versus identical placebo, up to 4 paired 10-day treatment periods, random order. Comparison of patient’s self-identified most troubling symptom using Likert scale assessed on 4 days of each treatment period.  **Control condition:** standard practice, all patients were instructed to stop theophylline and contact physician to resume treatment if dyspnea worsened. If symptoms improved, instructed to continue. |
| **Outcomes** | **Primary outcome:** quality of life and exercise capacity (change from baseline)  **Secondary outcomes:** proportion taking theophylline |
| **Trial status** | Published.  Mahon JL, Laupacis A, Hodder RV, McKim DA, Paterson NA, Wood TE, Donner A. Theophylline for irreversible chronic airflow limitation: a randomized study comparing n of 1 trials to standard practice. Chest. 1999 Jan;115(1):38-48. |
|  |  |
| **Mahon 1996** | |
| **Methods** | **Study design:** parallel group RCT  **Follow-up period:** 6 months  **Funding:** Ontario Ministry of Health, study medication supplied by pharmaceutical company  **Hypothesis:** objectivity of n-of-1 trials in determining treatment in a single patient would lead to a better outcome over standard practice, including the use of less medication |
| **Participants** | **Setting:** chronic airflow limitation clinic and the outpatient practice of a general physician  **Country:** Canada  **Population:** adults with chronic irreversible airflow limitation, history of use of theophylline in the preceding 1-5 years and uncertainty that it was helpful |
| **Interventions** | **N-of-1 trial:** double blinded comparison of theophylline versus identical placebo, up to 4 paired 10-day treatment periods, random order. Comparison of patient’s self identified most troubling symptom using Likert scale assessed on 4 days of each treatment period.  **Control condition:** standard practice, all patients were instructed to stop theophylline and contact physician to resume treatment if dyspnea worsened. If symptoms improved, instructed to continue. |
| **Outcomes** | **Primary outcome:** proportion of patients taking theophylline at 6 months  **Secondary outcomes:** quality of life, functional exercise capacity (change from baseline) |
| **Trial status** | Published.  Mahon J, Laupacis A, Donner A, Wood T. Randomised study of n of 1 trials versus standard practice. BMJ. 1996 Apr 27;312(7038):1069-74. |
|  |  |
| **Johnston 1993** | |
| **Methods** | **Study design:** parallel group RCT “matched in pairs on sex and age and randomly assigned” but method of randomization and allocation concealment are not described.  **Follow-up period:** 3 weeks for primary outcome  **Funding:** CIBA-GEIGY Canada (pharmaceutical industry)  **Hypothesis:** how the effectiveness of methylphenidate (MPH) is evaluated may alter how acceptable parents feel the medication is, how satisfied they are with the evaluation and treatment, and how willing they are to adhere to the subsequent recommendations regarding MPH use |
| **Participants** | **Setting:** outpatient child psychiatry unit of a teaching hospital  **Country:** Canada  **Population:** childhood attention-deficit hyperactivity disorder |
| **Interventions** | **N-of-1 trial:** comparing double blinded treatment with higher and lower doses of MPH versus identical placebo, randomly assigned across days by hospital pharmacy. Length of treatment periods and number of crossovers was not described. Comparison using daily assessment of child behavior (Conner’s Teacher’s scale) and additional measures of academic performance, peer relationships, and side effects.  **Control condition:** “typical clinical procedure” MPH dose chosen at resident’s discretion, weekly assessments of treatment effects by parents and teacher report. |
| **Outcomes** | **Primary outcome:** acceptability of treatment by Modified Treatment Evaluation Inventory  **Secondary outcomes:** parent satisfaction, multiple measures of adherence, number of patients prescribed methylphenidate |
| **Trial status** | Published.  Johnston C, Fine S. Methods of evaluating methylphenidate in children with attention deficit hyperactivity disorder: acceptability, satisfaction, and compliance. J Pediatr Psychol. 1993 Dec;18(6):717-30. |
